# Editorial: Spotlight on Solanaceae metabolism: Biotechnological application, volume II

**DOI:** 10.3389/fpls.2022.1102108

**Published:** 2022-12-16

**Authors:** Zsófia Bánfalvi, Amalia Barone, Glenn Bryan

**Affiliations:** ^1^Hungarian University of Agriculture and Life Sciences, Gödöllő, Hungary; ^2^University of Naples Federico II, Naples, Italy; ^3^The James Hutton Institute, Dundee, United Kingdom

**Keywords:** tomato, gene editing, green flesh locus, lycopene, beta-carotene, uridine diphosphate UDP-glycosyltransferase, biomanufacturing

The Solanaceae, or nightshade family, is one of the largest dicotyledonous plant families including nearly 2500 species with enormous morphological and chemical diversity. Many solanaceous species have fundamental economic importance as foods, feeds, herbs or ornaments. Potato (*Solanum tuberosum*), tomato (*S. lycopersicum*), eggplant (*S. melongena*) and pepper (*Capsicum annuum*) are just some of the most important crops that belong to this family. The total tomato production exceeds 187 million tonnes on 5 million hectares in 175 countries. These data highlight the importance of tomato in human diet and urge breeders and researchers to study and breed tomatoes with higher yield, quality and stress tolerance. This interest is reflected in the following three articles of the Research Topic.

The CRISPR/Cas9-mediated gene editing has already proved itself to be an efficient tool to generate mutants for research applications. However, there are few examples in literature in which the safety and robustness of a modification induced by gene editing have been extensively studied. This prompted Gianoglio et al. to study the editing efficiency of the CRISPR/Cas9 system in two different tomato cultivars, assess the potential off-target mutations, and evaluate the phenotype of edited lines carrying different mutated alleles. The experiments were focused on the *GREEN FLESH/STAYGREEN* (*GF*) locus, which encodes a Mg dechelatase required for chlorophyll catabolism. The results showed considerable efficiency and reproducibility when generating *gf* mutants through CRISPR/Cas9. Furthermore, the gene-edited lines closely recapitulated known features of tomato *gf* mutants obtained with traditional breeding techniques, while also shedding light on novel traits such as vitamin E overaccumulation and resistance to the gray mold, *Botrytis cinerea*, which is considered to be the most important pathogen responsible for postharvest decay of fresh fruits.

CRISPR/Cas9 tends to form mainly indels resulting in gene knockouts, which can be deleterious in certain cases. Target activation-induced cytidine deaminase (Target-AID) base-editing technology with the CRISPR/Cas9 system fused with activation-induced cytidine deaminase (AID) can avoid this problem as the use of this system results in base pair substitution. Hunziker et al. analysed allelic versions in tomato mutants that were obtained previously by Target-AID base-editing technology for their impact on plant growth and fruit development. In triple-substituted lines mutated in *DNA DAMAGE UV BINDING PROTEIN 1* (*SlDDB1*), *DE-ETIOLATED 1* (*SlDET1*) and *LYCOPENE-Β-CYCLASE* (*SlCYC-B*), higher accumulation of lycopene and β-carotene was observed than in the fruits of non-mutated control plants. Carotenoids play an important role in human nutrition because of their antioxidant and pro-vitamin A activities and anticancer potential. Thus, the base-edited lines have a potential for commercial application.

Pesticides are widely used to prevent crop losses and increase yield. Pesticide residues, however, seriously affect food safety. Plants can convert the pesticides into less toxic compounds. Uridine diphosphate UDP-glycosyltransferase (UGT) is one the enzymes involved in the detoxification process. There are hundreds of UGTs involved in different pathways in different plant species. However, the *UGT* gene family has not been characterised yet in tomato. This knowledge gap was diminished by Yu et al., who identified a total of 143 *UGT* genes in tomato with highly conserved gene structure and motives. The promoter region of *SlUGT* genes contain several binding sites for plant hormone- and stress responsive transcription factors and can be induced by glutathion and chlorothalonil, a widely used pesticide and fungicide. These results suggest that *UGT* genes in tomato possess similar functions to those in other plant species.

*Nicotiana* species also belong to the Solanaceae family and *N. benthamiana* can be applied as a production platform for high value compounds. “Sexy Plants” of *N. benthamiana* produce an insect sex pheromone based on the constitutive expression of three genes with different origins. Nevertheless, high level of pheromone synthesis led to growth retardation diminishing the potential for commercial use of “Sexy Plants” for biomanufacturing. Jutersek et al. were interested in the underlying molecular changes and studied the transcriptome of the plants producing the pheromone in different quantities. They found a tremendous transcriptional reprogramming in leaves of high-producing plants including stress-like responses accompanied with upregulation of jasmonic acid and downregulation of gibberellic acid signalling pathways, which may explain the retarded growth of the plants.

We thank the authors and reviewers for their valuable support.

## Author contributions

All authors listed have made a substantial, direct, and intellectual contribution to the work and approved it for publication.

